# Neuropsychiatric Outcome of Children Born to Women With Systemic Lupus Erythematosus and Exposed *in Utero* to Azathioprine: A Case-Control Study

**DOI:** 10.3389/fphar.2020.613239

**Published:** 2020-12-16

**Authors:** Maria-Grazia Lazzaroni, Laura Andreoli, Francesca Crisafulli, Francesco Tamborini, Irene Debeni, Valentina Binda, Cecilia Nalli, Jessica Galli, Elisa Fazzi, Gabriella Moroni, Franco Franceschini, Angela Tincani

**Affiliations:** ^1^ Department of Clinical and Experimental Sciences, University of Brescia, Brescia, Italy; ^2^ Rheumatology and Clinical Immunology Unit, ASST Spedali Civili of Brescia, Brescia, Italy; ^3^ Nephrology Unit, IRCCS Ca’ Granda Ospedale Maggiore Policlinico, Milano, Italy; ^4^ Neuropsychiatry Unit, Children’s Hospital, ASST Spedali Civili of Brescia, Brescia, Italy; ^5^I.M. Sechenov First Moscow State Medical University, Moscow, Russia

**Keywords:** azathioprine, offspring, neurodevelopmental disorders, learning disabilities, pregnancy, systemic lupus erythematosus

## Abstract

**Objective:** The long-term outcome of children born to SLE mothers still represents a controversial topic in literature, with some studies reporting a possible increased prevalence of different neurologic and psychiatric diseases (NPD), including neurodevelopmental disorders (ND), and in particular learning disorders (LD). Different risk factors have been advocated, such as the *in utero* exposure to auto-antibodies and drugs, particularly Azathioprine (AZA).

**Methods:** A case-control study was designed to compare pregnancies treated with AZA (cases) with those not treated with AZA (controls). All the pregnancies had been prospectively followed in two Italian centers. The match was based upon renal involvement, antiphospholipid (aPL) status, maternal age at pregnancy (±5 years) and child’s age at the time of the study (±2 years). SLE mothers were interviewed by a telephone survey, particularly focused on the presence of a certified NPD in their children ≥6 years of age.

**Results:** Twenty-seven cases and 65 controls were similar in terms of demographic, immunological and clinical features, except for a higher rate of SLE flares during pregnancy in cases (22.2% vs. 10.8%, *p*:0.191). The 92 children had a mean age of 14.0 years at the time of the survey; 11 had at least one NPD (12.0%). The frequency of each single NPD was similar to that of the general pediatric population and no association was found with either the *in utero* exposure to AZA, or other specific factors (auto-antibodies, disease activity, obstetric complications, prematurity).

**Conclusion:** The long-term neuropsychiatric outcome of the children born to SLE mothers did not show neither an increased frequency of NPD as compared to the general pediatric population nor a specific pattern of NPD. The *in utero* exposure to AZA was not associated with the development of NPD in this case-control study of prospectively-followed pregnancies. NPD are complex conditions and large prospective studies are needed to capture the wide range of variables that may contribute to their development in the offspring of SLE women.

## Introduction

Systemic Lupus Erythematosus (SLE) is an autoimmune disease mostly affecting women of childbearing age (Gergianaki et al., 2018). In the last decades, the rate of successful pregnancies for these women has greatly increased, thanks to the general improvement in SLE management, but also to the availability of drugs compatible with pregnancy and lactation (Lazzaroni et al., 2016). Pregnancy in SLE requires a multidisciplinary approach, with an accurate preconception counseling and a close monitoring, helping to reduce maternal flares and obstetric and neonatal complications (Andreoli et al., 2017). Besides the impact of the disease on pregnancy outcome, another topic is of utmost interest for patients who wish to become mothers: the short- and the long-term outcome of their children.

A recent large multicentre Italian survey, focused on the impact of SLE and other rheumatic diseases on family planning, revealed that most of these women were concerned about the impact of their disease on parenting capacity, but also the possible harmful effects of drugs assumed during pregnancy and lactation on the growth of their offspring (Andreoli et al., 2019). These concerns were often the reason why these women had fewer children than desired. The same study also revealed that patients had poor knowledge about reproductive issues, mainly because of the lack of discussion with health care providers.

Altogether, these data highlight the urgent need of an informative and exhaustive preconception counseling and a close monitoring during pregnancy by a multidisciplinary team, also encompassing the topic of “children’s health”.

Reassuring data about the short-term outcome of these children, in terms of neonatal complications and possible teratogenic effects of the drugs, have become available in the past few years. It is now needed to clarify the long-term outcome of the offspring of SLE mothers (Yengej et al., 2017). In fact, some studies raised the possibility of an increased frequency of different neuro-psychiatric disorders (NPD), including neurodevelopmental disorders (ND), such as learning disabilities (LD) and autism spectrum disorders (ASD), even if a normal intelligence level was reported (Urowitz et al., 2008; Nalli et al., 2020). Specifically, two case-control studies highlighted an increased frequency of LD in the offspring of SLE patients, particularly regarding male children (McAllister et al., 1997; Ross et al., 2003). More recently, a large population study based on the OSLER registry (Offspring of SLE Mothers Registry) found an increased risk of ASD, even though the overall prevalence was very low in absolute terms (Vinet et al., 2015).

In order to explain this increased frequency of NPD, different potential risk factors have been advocated. Apart from general social and familiar risk factors, such as coping with a mother affected by a chronic disease, specific SLE-related factors have been considered. In particular, the transplacental passage of maternal autoantibodies and/or drugs could be responsible for NPD by crossing the incomplete blood-brain barrier and binding to neuronal cells in the developing central nervous system of the fetus (Nalli et al., 2020).

The presence of anti-Ro/SSA antibodies was reported to be associated with an increased risk of LD in children of SLE patients (Ross et al., 2003; Urowitz et al., 2008). However, a study evaluating the neurodevelopmental outcome in 13 children born with congenital heart block, a disease which is strongly associated with these antibodies, found a normal intelligence quotient and only one case of a mild LD (Brucato et al., 2006). Antiphospholipid antibodies (aPL) have been associated with NPD (Motta et al., 2006), including ND and behavioral disorders, but also with increased usage of special educational services (Abisror et al., 2013; Mekinian et al., 2013; Marder et al., 2014).

Regarding the potential role of drugs commonly prescribed in SLE pregnancies, special attention was raised on Azathioprine (AZA) and its metabolites (Belizna et al., 2020). AZA is an immunosuppressive drug widely used in SLE patients, especially to maintain remission, and is considered safe during pregnancy as supported by international guidelines (Skorpen et al., 2016; Andreoli et al., 2017).

As maternal treatment is crucial for keeping the disease under control and favoring a good pregnancy outcome, it is important to achieve the patient’s compliance by providing reassuring data about the use of drugs during pregnancy. Therefore, we designed a case-control study to elucidate the effects of *in utero* exposure to AZA in children born to women with SLE.

## Materials and Methods

### Study Design

The present study was designed as a retrospective case-control study including pregnancies of SLE patients prospectively followed in two Italian centers (Brescia, Milano). SLE pregnancies treated with AZA formed the group of cases, while SLE pregnancies not treated with AZA formed the control group. The match between one case with two or, if possible three controls, was based on the subsequent characteristics: renal involvement (present/absent); aPL status at the beginning of pregnancy (positive/negative); age of the mother at the beginning of pregnancy (±5 years); age of the child at the time of the study (±2 years).

The study was performed according to the principles of the Declaration of Helsinki and was approved by all the Local Ethic Committees (approval number 2917 in the Promoting Center). All patients signed a written informed consent.

### Inclusion and Exclusion Criteria

Women with SLE diagnosis fulfilling the 1997 ACR classification criteria (Hochberg, 1997) were considered if they had a singleton pregnancy yielding a live birth. Only children of school age (≥6 years) at the time of interview were included in the study.

Twin Pregnancies and pregnancy losses defined as spontaneous abortion (<10th gestational weeks) or intrauterine fetal death (≥10th gestational weeks) were excluded.

### Clinical and Immunological Features of Mothers and Children

Data regarding clinical and laboratory features were retrospectively retrieved from medical charts from the two centers and collected in a common database.

The following maternal features were collected: age at disease onset, age at the beginning of pregnancy; disease duration at the beginning of pregnancy; presence of SLE renal involvement; occurrence and type of disease flare during pregnancy; therapy prescribed during pregnancy, including Hydroxychloroquine (HCQ), AZA, Cyclosporine or other immunosuppressants, corticosteroids as mean weekly dosage of prednisone equivalent; occurrence of pregnancy complications.

### Neonatal Features Collected Were Gestational Week at Birth, Birth Weight and Sex

The immunological tests were performed during pregnancy in each of the two participating centers, by certified laboratories fulfilling European quality standards. Antinuclear antibodies (ANA), antibodies to extractable nuclear antigens (anti-ENA) and aPL were detected as for clinical practice. A complete aPL profile composed by the three criteria assays was available for each subject; each assay was considered positive if confirmed at least 12 weeks apart.

### Long-Term Neuropsychiatric Outcome of Children

The long-term neuropsychiatric outcome of the children was assessed through a specifically designed telephone interview in a time interval comprised between May 2019 and May 2020. Questions concerned the presence of different neurologic and psychiatric disorders (NPD) and included the subsequent diagnosis:

- Neurodevelopmental Disorders (ND): Attention-Deficit/Hyperactive Disorder (ADHD), Learning Disabilities (LD) including dyslexia, dysgraphia, dyscalculia, dysorthography, Communication Disorders, Autism Spectrum Disorder (ASD);
- Psychomotor delay;
- Behavioral disorders including aggressiveness, depression/anxiety, hyperactivity;
- Other neurological disorders: epilepsy; migraine.

Only disorders which had been certified by a child neurologist or psychiatrist were considered. Information regarding the repetition of school years was also collected.

### Statistical Analysis

Continuous variables were reported as mean value and standard deviation, whereas categorical variables as proportion and/or percentage. T-student’s test for continuous variables and Fisher’s exact test or Chi-square test for categorical variables were applied as appropriate. *p* values < 0.05 were considered significant.

## Results

### Description of the Cohort

#### Pregnancies Characteristics

The study included 92 singleton pregnancies resulted in live births of 76 SLE patients from two Italian referral centers (Brescia n = 43, Milano n = 33) in a period comprised between 1987 and 2014. Fourteen patients had two pregnancies and one patient had three pregnancies. Most of the patients was Caucasian (97.4%), while the remaining two patients were Asian (2.6%). Mean age at the onset of SLE was 23.1 ± 6.5 years and mean disease duration at the beginning of pregnancy was 9.2 ± 6.0 years; renal involvement was present in 50/76 (65.8%). An associated antiphospholipid syndrome (APS) was detected in 11/76 (14.5%) patients and autoimmune thyroiditis in 7.9%.

At the beginning of pregnancy, the mean age of the mothers was 32.3 ± 4.9 years. A SLE flare during pregnancy occurred in 13/92 (14.1%): nine of 13 total flares were renal (9.8%), three were cutaneous (3.3%), one neurologic and one articular.

Regarding the immunological profile, anti-ENA antibodies were positive in 54 pregnancies (58.7%) and 27 of them were positive for anti-Ro/SSA antibodies (29.3%). aPL positivity was detected in 26.1% of pregnancies: 6.5% had a triple aPL positivity (LAC plus aCL IgG/IgM plus aB2GPI IgG/IgM), 7.6% had double aPL positivity and 11.9% had a single positivity. Only two patients with two pregnancies each had an aPL profile modification between the first and the second pregnancy: in one patient a double positivity turned into negative, while the other patient who was previously aPL negative displayed a single positivity.

Preterm delivery <37th weeks was recorded in 25/92 pregnancies (27.2%), including six preterm deliveries <34th weeks. Pre-eclampsia occurred in 8/92 (9.7%) of pregnancies, while HELLP in 1/92 (1.1%).

Most of the patients were treated with corticosteroids during pregnancy (78.2%), with a mean weekly prednisone dosage of 31.5 mg, while hydroxychloroquine (HCQ) was prescribed in half of the pregnancies (51.1%).

#### Children’s Characteristics

The mean age of the 92 children was 14.0 ± 6.2 years (range: 6–33 years); 47.8% were males. Mean gestational age at birth was 37.0 ± 2.1 weeks and mean birth weight was 2865.3 ± 540.2 g. Small for Gestational Age (SGA) babies were 7/89 (7.9%).

On the basis of the telephone survey, we were able to identify 11 out of 92 children (12.0%) with at least one diagnosis of NPD, as certified by a specialist. One child had epilepsy, one had psychomotor delay, while the other nine had at least one diagnosis of ND; three children had more than one type of NPD. Overall, 11 different diagnosis of ND were recorded: three communication disorders (expressive language delay, 3.3%), two ADHD (2.2%) and six LD (6.5%). Among LD, the most frequent diagnosis was dyslexia, reported in five children (5.4%), including one child with concomitant dyscalculia, dysgraphia and dysorthography (1.1%). The only LD child without dyslexia had dyscalculia and dysgraphia. No cases of ASD were found.

Two cases of behavioral disorders were collected: 1 case of hyperactivity and 1 case of aggressiveness, together with psychomotor delay. The other two children with multiple NPD had expressive language disorder with dyslexia.

Moreover, we recorded two children with migraine (all the 3 with other concomitant ND or LD).

### Case-Control Study

A univariate analysis was performed to compare 27 pregnancies of 22 patients treated with AZA (cases) with 65 matched pregnancies of 56 patients not treated with AZA during pregnancy (controls) (Table 1). Two patients had two pregnancies each, one treated with AZA and one not treated with AZA; therefore, the apparent total number of patients is 78.

**TABLE 1:** Univariate analysis of the case-control study comparing SLE pregnancies treated with AZA (cases; n patients = 22, n pregnancies = 27) and not treated with AZA (controls, n patients = 56, n pregnancies = 65).

	Pregnancies treated with AZA	Pregnancies not treated with AZA	*p*-value
**Patients characteristics**			
Age at SLE onset (years), *mean (SD)*	23.7 (6.7)	22.9 (6.4)	0.610
Caucasian ethnicity	21/22 (95.5)	55/56 (98.2)	0.487
Renal involvement	15/22 (68.2)	37/56 (66.1)	1.000
Concomitant autoimmune diseases	11/22 (50.0)	22/56 (39.3)	0.450
Antiphospholipid syndrome	5/22 (22.7)	6/56 (10.7)	0.276
Sjӧgren’s syndrome	1/22 (4.6)	2/56 (3.6)	1.000
Rheumatoid arthritis	0/22 (0.0)	1/56 (1.8)	1.000
Autoimmune thyroiditis	1/22 (4.6)	5/56 (8.9)	0.670
**Pregnancies characteristics**			
Age at pregnancy (years), mean (SD)	32.7 (5.9)	32.1 (4.4)	0.565
Disease duration at the beginning of pregnancy (years), mean (SD)	9.07 (6.7)	9.20 (5.8)	0.719
Disease flare during pregnancy	6/27 (22.2)	7/65 (10.8)	0.191
Renal	2/27 (7.4)	7/65 (10.8)	1.000
Cutaneous	3/27 (11.1)	0/65 (0.0)	0.023
Neurologic	1/27 (3.7)	0/65 (0.0)	0.294
Articular	1/27 (3.7)	0/65 (0.0)	0.294
Antiphospholipid antibodies positivity	9/27 (40.7)	15/65 (27.7)	0.311
Triple positivity	1/27 (3.7)	5/65 (7.7)	0.667
Double positivity	4/27 (14.8)	3/65 (4.6)	0.188
Single positivity	4/27 (14.8)	7/65 (10.8)	0.725
Anti-ENA positivity	17/27 (63.0)	38/65 (58.5)	0.816
Anti-Ro/SSA positivity	7/27 (25.9)	21/65 (32.3)	0.625
pPROM (<37th weeks)	1/27 (3.7)	5/65 (7.7)	0.667
HELLP syndrome	0/27 (0.0)	1/65 (1.5)	1.000
Preeclampsia	2/27 (7.4)	6/65 (9.2)	1.000
**Treatment during pregnancy**			
Corticosteroids	26/27 (96.3)	47/65 (72.3)	0.010
Weekly dose of steroids in prednisone equivalent	39.7 (37.3)	26.9 (27.2)	0.128
Hydroxychloroquine	14/27 (51.9)	33/65 (50.8)	1.000
Cyclosporine-A	0/27 (0.0)	9/65 (13.9)	0.054

HELLP, hemolysis, elevated liver enzymes, and low platelets; pPROM, preterm premature rupture of membranes. Two patients had two pregnancies each, one treated with AZA and one not treated with AZA; therefore, the apparent total number of patients is 78. Results are presented as n/n available data (%), except otherwise indicated.

The two groups did not differ in terms of frequency of renal involvement, aPL positivity, maternal age at the beginning of pregnancy and children age at the time of the interview. These were the characteristics *a priori* selected for matching the two groups. The distribution of aPL profiles (single, double, triple aPL profile according to the number of positive tests) was also homogenous in the two groups. Other clinical and laboratory features were also similar between cases and controls: age at SLE onset and disease duration at the beginning of pregnancy, prevalence of Caucasian ethnicity and other concomitant autoimmune disorders (especially autoimmune thyroiditis) and rate of anti-Ro/SSA positivity.

The frequency of disease flare was higher in cases than in controls, although not statistically significant (22.2% vs. 10.8%, *p*:0.191); flares were mostly renal in both groups (7.4% vs. 10.8%; *p*:1.000). Three cutaneous, one neurologic and one articular flare were also observed in the AZA group, while none in the control group.

Regarding therapy during pregnancy, corticosteroids use was more frequent in the AZA group (96.3% vs. 72.3% *p*: 0.010), with a higher mean weekly prednisone-equivalent dosage as compared to controls, although not statistically significant (39.7 ± 37.3 vs. 26.9 ± 27.2 mg, *p*: 0.128). Hydroxychloroquine was prescribed to half of the patients during pregnancy in both groups (51.9% vs. 50.8%; *p*: 1.000).

Among pregnancies not treated with AZA, 9 (13.9%) were treated with Cyclosporine (including 3 with concomitant HCQ), while 30 only with HCQ (46.2%) and 15 only with corticosteroids (23.1%). Eleven patients (16.9%) were not taking any immunosuppressive drugs or corticosteroids during pregnancy.

Considering the 92 children born to the above described 92 pregnancies from 76 SLE mothers (Table 2), the mean age at the time of the interview was 12.3 ± 4.9 years in cases and 14.7 ± 6.6 in controls (*p*: 0.165). Male/female ratio, birth weight and gestational age at birth were also similar in the two groups, as well as the rate of prematurity (<37th weeks) or severe prematurity (<34th weeks).

**TABLE 2:** Univariate analysis of the case-control study comparing the outcome of children born to SLE pregnancies included in the study and exposed *in utero* to AZA (n children = 27) vs. not-exposed *in utero* to AZA (n children = 65).

	Children exposed to AZA *in utero*	Children not exposed to AZA *in utero*	*p*-value
Age at the time of the interview, mean (SD)	12.3 (4.9)	14.7 (6.6)	0.165
Male sex	14/27 (51.9)	30/65 (46.2)	0.653
Birth weight (g), mean (SD)	2798.0 (550.2)	2891.6 (538.4)	0.304
Gestational age at birth, mean (SD)	36.8 (2.5)	37.1 (2.0)	0.904
Small for gestational age	0/25 (0.0)	7/64 (10.9)	0.101
Preterm birth (<37th weeks)	8/27 (29.6)	17/65 (26.2)	0.799
Severe preterm birth (<34th weeks)	3/27 (11.1)	3/65 (4.6)	0.354
At least one NPD	3/27 (11.1)	8/65 (12.3)	1.000
ND	2/27 (7.4)	12/65 (18.5)	0.219
ADHD	1/27 (3.7)	1/65 (1.5)	0.503
Communication disorders	1/27 (3.7)	2/65 (3.1)	1.000
Learning disabilities	0/27 (0.0)	6/65 (9.2)	0.175
Dyslexia	0/27 (0.0)	5/65 (7.7)	0.317
Dyscalculia	0/27 (0.0)	2/65 (3.1)	1.000
Dysgraphia	0/27 (0.0)	2/65 (3.1)	1.000
Dysorthography	0/27 (0.0)	1/65 (1.5)	1.000
Behavioral disorders	1/27 (3.7)	1/65 (1.5)	0.502
Psychomotor delay	0/27 (0.0)	1/65 (1.5)	1.000
Epilepsy	1/27 (3.7)	0/65 (0.0)	0.294
Migraine	0/27 (0.0)	2/65 (3.1)	1.000
Repetition of school years	1/27 (3.7)	4/65 (6.2)	1.000

LD, Learning Disabilities; ND, Neurodevelopmental Disorders; NPD, Neuro-Psychiatric Disorders. Results are presented as n/n available data (%), except otherwise indicated.

There was no difference in terms of prevalence of NPD, both considering the global prevalence of all the diseases and the single diagnosis. The frequency of ND, in particular LD, and the repetition of school years, was lower in children exposed *in utero* to AZA, although without a statistically significant difference.

### Comparison of Children With and Without Neurodevelopmental Disorders/Learning Disorders

An additional univariate analysis was performed to compare children with at least one diagnosis of NPD (n = 11) and children without any diagnosis of NPD (n = 81) (Table 3), in order to identify possible factors associated with these disorders.

**TABLE 3:** Univariate analysis comparing children with NPD (n = 11) to children without NPD (n = 81), born to the 92 SLE pregnancies included.

	NPD present	NPD absent	*p*-value
**Maternal characteristics**			
Age at SLE onset (years), mean (SD)	22.1 (6.5)	23.3 (6.5)	0.745
Caucasian ethnicity	11/11 (100.0)	80/81 (93.8)	1.000
Renal involvement	7/11 (63.6)	57/81 (70.4)	0.730
Concomitant autoimmune diseases	7/11 (63.6)	30/81 (37.0)	0.110
Antiphospholipid syndrome	3/11 (36.4)	9/81 (11.1)	0.721
Sjӧgren’s syndrome	1/11 (9.1)	8/81 (9.9)	1.000
Rheumatoid arthritis	0/11 (0.0)	1/81 (1.2)	1.000
Autoimmune thyroiditis	1/11 (9.1)	7/81 (8.6)	1.000
**Pregnancies characteristics**			
Age at pregnancy (years), mean (SD)	31.8 (3.2)	32.20 (5.0)	0.762
Disease duration at the beginning of pregnancy (years), mean (SD)	9.3 (5.6)	9.15 (6.1)	0.942
Disease flare during pregnancy	0/11 (0.0)	13/81 (16.0)	1.000
Renal	0/11 (0.0)	9/81 (11.1)	1.000
Cutaneous	0/11 (0.0)	3/81 (3.7)	1.000
Neurologic	0/11 (0.0)	1/81 (1.2)	1.000
Articular	0/11 (0.0)	1/81 (1.2)	1.000
Antiphospholipid antibodies positivity	3/11 (36.4)	21/81 (25.9)	1.000
Triple positivity	2/11 (18.2)	4/81 (4.9)	0.150
Double positivity	0/11 (0.0)	7/81 (8.6)	1.000
Single positivity	1/11 (9.1)	10/81 (12.3)	0.684
Anti-ENA positivity	8/11 (72.7)	46/81 (56.8)	0.347
Anti-Ro/SSA positivity	2/11 (18.2)	25/81 (30.9)	0.498
**Maternal treatment during pregnancy**			
Corticosteroids	8/11 (72.7)	64/81 (79.0)	0.285
Azathioprine	3/11 (36.4)	24/81 (29.6)	1.000
Hydroxychloroquine	8/11 (72.7)	39/81 (48.1)	0.511
Cyclosporine-A	2/11 (18.2)	7/81 (8.74)	0.333
**Children’s characteristics**			
Male sex	7/11 (63.6)	37/81 (45.7)	0.541
Birth weight (g), mean (SD)	2972.7 (389.8)	2850.1 (558.5)	0.933
Gestational age (weeks), mean (SD)	37.92 (2.2)	36.84 (2.2)	0.336
Small for gestational age	2/10 (20.0)	5/79 (6.3)	0.176
Preterm birth (<37th weeks)	0/11 (0.0)	25/81 (30.9)	0.169
Severe preterm birth (<34th weeks)	0/11 (0.0)	6/81 (7.4)	0.579

NPD, neuropsychiatric disorders. Results are presented as n/n available data (%), except otherwise indicated.

The two groups did not significantly differ in terms of maternal disease characteristics or pregnancy features, including specific drugs or maternal autoantibodies exposure during pregnancy. The neonatal features were also similar in the two groups, including weight and gestational age at birth.

## Discussion

Systemic Lupus Erythematosus (SLE) is a systemic autoimmune disorder, more frequently affecting women in their childbearing age and consequently having a significant impact on family planning and reproductive aspects (Gergianaki et al., 2018). The modifications of their health status determined by pregnancy, the increased risk of obstetric complications, as well as the children outcome are topic of crucial interest for these patients (Andreoli et al., 2017).

The long-term outcome of children born to SLE mothers still represents a controversial topic in literature, with some studies reporting a possible increased prevalence of different neurologic and psychiatric diseases (NPD), including neurodevelopmental disorders (ND), and in particular learning disorders (LD) (Yengej et al., 2017; Nalli et al., 2020). In order to explain this possible increased frequency of NPD in these children, different possible risk factors have been implicated, such as auto-antibodies and drugs assumed during pregnancy and Azathioprine (AZA). A recent literature review regarding therapy with AZA during pregnancy concluded that it does not seem to increase the risk of congenital abnormalities in the offspring, but more long-term studies are needed to analyze the association with NPD in children exposed *in utero*, while considering all the potential confounding factors including maternal disease and familiar, social and economic context (Belizna et al., 2020).

In particular, one study involving 60 children (age range: 3.4–9.2 years) born to 38 SLE mothers found an association between AZA treatment during pregnancy and special education requirement in the offspring: seven out of 13 children exposed *in utero* to AZA required special educational services. This support was requested mainly for language delays (23.3%), dyslexia (5%) and ADHD (5%) (Marder et al., 2013). Another study showed normal intelligence levels and neurological physical examination in a cohort of 40 children born to mothers with positive aPL and a diagnosis of SLE and/or APS, systematically evaluated by a child neurology-psychiatrist (Nalli et al., 2017). A diagnosis of LD was present in 19% of school-aged children, all born to mothers with positive aPL with three positive tests (triple aPL profile); none of seven children exposed *in utero* to AZA had ND or LD. Moreover, in a recent multicentre Italian survey involving women in reproductive age with different rheumatic diseases, no association was found between the reported presence of children NPD and *in utero* exposure to maternal auto-antibodies or drugs, including AZA (Lazzaroni et al., 2017).

The present study was designed as case-control and the instrument to collect data concerning the long-term neuropsychiatric outcome of the children was a telephone survey, in which SLE mothers were asked to report diagnosis of NPD only when certified by a pediatric neurology or psychiatrist. The two groups of pregnancies were matched in terms of renal involvement, immunologic profile (especially aPL), maternal age at pregnancy and age of the children at the time of the study. Considering the possible influence of all these factors on the gestational outcome and health status of the children born from these pregnancies, the homogeneity of these two groups allowed to evaluate the effects of *in utero* exposure to AZA as an independent factor.

Moreover, the mean age of the 92 children at the time of the study was 14.0 ± 6.20 years, significantly higher as compared to previous studies. This is very relevant to the correct estimation of the frequency of NPD and in particular LD, that can be diagnosed with certainty only during school years (Association, 2013).

In our cohort of 92 children, 11 cases of NPD were observed: 3/27 among those exposed to AZA and 8/65 among those not exposed to AZA, without any significant difference between the two groups (11.1% vs. 12.3%; *p*-value: 1.000). The frequency of ND and particularly LD, was higher in children not exposed to AZA, although without statistically significant difference.

Pregnancies in the group of cases and controls were comparable in terms of different characteristics, including the frequency of obstetric complications and particularly of preterm birth (<37th weeks) and severe preterm birth (<34th weeks), as well as the immunological profile (rate of anti-Ro/SSA and aPL positivity) and frequency of total flares, including renal flares. Only the frequency of cutaneous flares and corticosteroids use was higher in pregnancies treated with AZA as compared to controls, with a higher mean corticosteroid dosage, although not statistically significant (39.7 vs. 26.9 mg of weekly prednisone-equivalent dosage, *p*: 0.128).

By comparing the 11 children with NPD with the other 81 without, we did not observe any difference in terms of maternal disease, pregnancy complication or treatment, nor the neonatal outcome of the children. Importantly, we did not observe an association between prematurity and NPD and the rate of preterm delivery reported in our cohort (27.2%) reflects what previously reported by literature on SLE pregnancies (23–28%) (Lazzaroni et al., 2016).

Regarding the specific diagnosis of NPD, none of the children included in the study showed the presence of autism spectrum disorders (ASD), although a previous study large reported a higher prevalence in 719 SLE offspring as compared to 8,493 matched controls (1.4% [95% confidence interval (95% CI) 0.8–2.5] vs. 0.6% [95% CI 0.5–0.8]) (Vinet et al., 2015).

In our cohort the frequency of ADHD (2/92, 2.17%) was similar to that previously reported in a systematic review (1–5%) (Yengej et al., 2017), and even lower than that reported in general pediatric population (7.2% according to a recent meta-analysis) (Thomas et al., 2015). Concerning the language delay, the frequency was also lower than that reported in the general pediatric population in Italy (3–4%) (Cipriani et al., 2002), while previous data in children born to SLE mothers are lacking.

The frequency of LD certified by children neurologists and psychiatrist is 6.52% in this study, that is only slightly higher than that estimated in the general pediatric population (around 4.5% in children in scholar age), and lower than that previously reported in the offspring of SLE mothers (21.4–26%) (Yengej et al., 2017). Dyslexia was the most frequent LD, with a frequency similar to the general population (2.5–5%) and inferior to what previously reported for SLE offspring (14.3–21.6%) (Yengej et al., 2017). No cases of LD were observed in the group of children exposed *in utero* to AZA.

Epilepsy was also less frequent (1/92, 1.1%) then previously reported in children born to SLE and/or APS patients in a multidisciplinary study (10%) (Nalli et al., 2017), and similar to general Italian pediatric population in which it was estimated around 3% (Banerjee et al., 2009), although the relatively small sample size in our study does not allow to adequately compare this prevalence.

Altogether, these data suggest that the long-term neuropsychiatric outcome of the children born to SLE mothers seems to be reassuring, with an estimated frequency of NPD (certified by expert clinicians) similar to the general pediatric population. Only LD showed a slightly increased frequency as compared to general pediatric population. Importantly, *in utero* exposure to AZA does not seem to be associated with the development of NPD. Moreover, no other specific risk factors associated with these pathological outcomes emerged from this study, including autoantibodies, disease activity during pregnancy, obstetric complications or prematurity.

Regarding the role of autoantibodies, particularly aPL, fetal brain was suggested to be one of the main target organs of aPL in APS-mice (Bertolaccini et al., 2016). In our cohort the presence of aPL was relatively low (26.1%), even if equally distributed between cases and controls. Moreover, when we compared the two groups of children with NPD to those without NPD, we found no significant difference in terms of aPL exposure, even if a slightly increased frequency of triple aPL positivity was present in the group of affected children.

The limitations of the present study are: 1) the relatively small sample size, although derived from the historical cohorts of two referral centers for SLE; 2) a wide time interval (1987–2014) during which the management of SLE pregnancies has evolved and improved; on the other hand, we should consider that this factor was normalized by the match of cases and controls by children’s age; 3) the use of a telephone survey, implying the possibility of a recall bias especially for older pregnancies, even if only diagnosis certified by neuropsychiatrists were considered.

Moreover, it should be underlined that is difficult to demonstrate the association of one single drug with the development of NPD, which are very complex conditions with a multifactorial pathogenesis. Particularly, we cannot exclude that other factors not considered in our analysis cohort could have contributed to NPD, such as socio-demographic and educational levels or the exposure to other medications not specifically related to SLE. This is the case for example of acetaminophen, a drug that has been traditionally considered as safe during pregnancy, so that pregnant patients may miss to report its use to their Physicians. However, a study published in 2016 highlighted the possibility that children exposed to acetaminophen prenatally could be at increased risk of multiple behavioral difficulties (Stergiakouli et al., 2016). In our cohort, even if the use of acetaminophen was not systematically recorded, we can assume that the chance for a continuous use of this drug is rather low, since only one patient had an articular flare during pregnancy and was treated with corticosteroids.

In conclusion, this case-control study of children born to SLE patients, who had been prospectively followed during pregnancy, did not highlight any specific pattern of NPD, both in children exposed and not exposed to AZA. The practical message is very relevant to the clinicians who take care of SLE patients during childbearing age. At this moment, there are no solid data supporting an association between the use of AZA during pregnancy and the development of NPD in exposed children. Therefore, there is no need to give up AZA as therapeutic choice during pregnancy, as there are, on the other hand, data supporting it as a key-drug in the management of SLE pregnancies of patients with previous severe organ involvement (e.g., renal, neurologic) to minimize the risk of flare (Skorpen et al., 2016; Andreoli et al., 2017; Sammaritano et al., 2020). Large multicentre prospective studies are needed to confirm these findings and to explore other factors contributing to NPD, particularly those not specifically related to SLE.

Anyway, it is important to inform SLE mothers about possible symptoms of NPD in their children, and in particular LD, and to invite them to seek for a specialist evaluation in case of doubts. A chronic disabling condition during childhood and/or youth can have a major impact on family life and global children’s health, therefore early diagnosis and treatment are crucial. The inclusion of a child Psychiatrist-Neurologist in the multidisciplinary team dedicated to SLE patients and their families could be proposed as a future perspective for the holistic management of these young women.

## Data Availability Statement

The raw data supporting the conclusions of this article will be made available by the authors, without undue reservation.

## Ethics Statement

The studies involving human participants were reviewed and approved by Comitato Etico di Brescia. The patients/participants provided their written informed consent to participate in this study.

## Author Contributions

M-GL, LA, AT, GM, and FF designed the study. M-GL, LA, CN, and ID organized the database. M-GL, LA, AT, CN, FC, ID, FT, VB, GM, and FF evaluated the patients and fulfilled the database. M-GL, ID, FC, LA, AT, EF, JG, FT, VB, and JM wrote the manuscript. All authors reviewed the manuscript draft, read and approved the submitted version.

## Conflict of Interest

The authors declare that the research was conducted in the absence of any commercial or financial relationships that could be construed as a potential conflict of interest.

The handling editor declared a past co-authorship with several of the authors LA and AT.

## References

[B1] Abisror N. Mekinian A. Lachassinne E. Nicaise-Roland P. De Pontual L. Chollet-Martin S. (2013). Autism spectrum disorders in babies born to mothers with antiphospholipid syndrome. Semin. Arthritis Rheum. 43 (3), 348–351. 10.1016/j.semarthrit.2013.07.001 23910451

[B2] Andreoli L. Crisafulli F. Tincani A. (2017) Pregnancy and reproductive aspects of systemic lupus erythematosus. Curr. Opin. Rheumatol. 29, 473–479. 10.1097/BOR.0000000000000415 28678065

[B3] Andreoli L. Lazzaroni M. G. Carini C. Dall’Ara F. Nalli C. Reggia R. (2019). “Disease knowledge index” and perspectives on reproductive issues: a nationwide study on 398 women with autoimmune rheumatic diseases. Joint Bone Spine 86, 475–481. 10.1016/j.jbspin.2018.12.002 30579917

[B4] Association, A. P. (2013). Diagnostic and statistical manual of mental disorders (dsm-5®). American Psychiatric Pub. 10.1590/s2317-17822013000200017 24413388

[B5] Banerjee P. N. Filippi D. Hauser W. A. (2009). The descriptive epidemiology of epilepsy—a review. Epilepsy Res. 85, 31–45. 10.1016/j.eplepsyres.2009.03.003 19369037 PMC2696575

[B6] Belizna C. Meroni P. L. Shoenfeld Y. Devreese K. Aliotaj-Reig J. Esteve-Valverde E. (2020). Utero exposure to azathioprine in autoimmune disease. Where do we stand? Autoimmun. Rev. 19 (9), 102525 10.1016/j.autrev.2020.102525 32240856

[B7] Bertolaccini M. L. Contento G. Lennen R. Sanna G. Blower P. J. Ma M. T. (2016). Complement inhibition by hydroxychloroquine prevents placental and fetal brain abnormalities in antiphospholipid syndrome. J. Autoimmun. 75, 30–38. 10.1016/j.jaut.2016.04.008 27160365 PMC6203312

[B8] Brucato A. Astori M. G. Cimaz R. Villa P. Destri M. L. Chimini L. (2006). Normal neuropsychological development in children with congenital complete heart block who may or may not be exposed to high-dose dexamethasone in utero. Ann. Rheum. Dis. 65, 1422–1426. 10.1136/ard.2005.049866 16504990 PMC1798357

[B9] Cipriani P. Chilosi A. Pfanner L. Villani S. Bottari P. (2002). Il ritardo del linguaggio in età precoce: profili evolutivi ed indici di rischio. O Capirci, MC Caselli (a cura di), Indici di rischio nel primo sviluppo del linguaggio: ricercar, clinica. Educazione, 95–108.

[B10] Gergianaki I. Bortoluzzi A. Bertsias G. (2018). Update on the epidemiology, risk factors, and disease outcomes of systemic lupus erythematosus. Best Pract. Res. Clin. Rheumatol. 32, 188–205. 30527426 10.1016/j.berh.2018.09.004

[B11] Hochberg M. C. (1997). Updating the american college of rheumatology revised criteria for the classification of systemic lupus erythematosus. Arthritis Rheum. 40, 1725 10.1002/1529-0131(199709)40:9<1725::AID-ART29>3.0.CO;2-Y 9324032

[B12] Lazzaroni M. G. Dall’Ara F. Fredi M. Nalli C. Reggia R. Lojacono A. (2016). A comprehensive review of the clinical approach to pregnancy and systemic lupus erythematosus. J. Autoimmun. 74, 106–117. 10.1016/j.jaut.2016.06.016 27377453

[B13] Lazzaroni M. G. Nalli C. Andreoli L. Carini C. Rodrigues M. Dall’Ara F. (2017). Long-term follow-up of 320 children born to mothers with systemic autoimmune diseases: a multicentre survey from 24 rheumatology centers in Italy. Arthritis Rheumatology S69.

[B14] Marder W. Ganser M. A. Romero V. Hyzy M. A. Gordon C. McCune W. (2013). Utero azathioprine exposure and increased utilization of special educational services in children born to mothers with systemic lupus erythematosus. Arthritis Care Res. 65, 759–766. 10.1002/acr.21888 PMC3572294 23139238

[B15] Marder W. Romero V. C. Ganser M. A. Hyzy M. A. Gordon C. McCune W. (2014). Increased usage of special educational services by children born to mothers with systemic lupus erythematosus and antiphospholipid antibodies. Lupus Sci. Med. 1 10.1136/lupus-2014-000034 PMC4213825 25379194

[B16] McAllister D. L. Kaplan B. J. Edworthy S. M. Martin L. Crawford S. G. Ramsey-Goldman R. (1997). The influence of systemic lupus erythematosus on fetal development: cognitive, behavioral, and health trends. J. Int. Neuropsychol. Soc. 3, 370–376. 10.1017/s1355617797003706 9260446

[B17] Mekinian A. Lachassinne E. Nicaise-Roland P. Carbillon L. Motta M. Vicaut E. (2013). European registry of babies born to mothers with antiphospholipid syndrome. Ann. Rheum. Dis. 72, 217–222. 10.1136/annrheumdis-2011-201167 22589374 PMC3551221

[B18] Motta M. Chirico G. Rebaioli C. B. Faden D. Lojacono A. Allegri F. (2006). Anticardiolipin and anti-beta2 glycoprotein i antibodies in infants born to mothers with antiphospholipid antibody-positive autoimmune disease: a follow-up study. Am. J. Perinatol. 23, 247–252. 10.1055/s-2006-939533 16625500

[B19] Nalli C. Galli J. Lazzaroni M. G. Andreoli L. Fazzi E. Tincani A. (2020). Long-term outcome of children born from mothers with autoimmune diseases. Best Pract. Res. Clin. Obstet. Gynaecol. 64, 107–116. 10.1016/j.bpobgyn.2019.11.003 31787531

[B20] Nalli C. Iodice A. Andreoli L. Galli J. Lojacono A. Motta M. (2017). Long-term neurodevelopmental outcome of children born to prospectively followed pregnancies of women with systemic lupus erythematosus and/or antiphospholipid syndrome. Lupus 26, 552–558. 10.1177/0961203317694960 28394231

[B21] Ross G. Sammaritano L. Nass R. Lockshin M. (2003). Effects of mothers’ autoimmune disease during pregnancy on learning disabilities and hand preference in their children. Arch. Pediatr. Adolesc. Med. 157, 397–402. 10.1001/archpedi.157.4.397 12695238

[B22] Sammaritano L. R. Bermas B. L. Chakravarty E. E. Chambers C. Clowse M. E. Lockshin M. D. (2020). American College of Rheumatology guideline for the management of reproductive health in rheumatic and musculoskeletal diseases. Arthritis Care Res. 72, 461–488. 10.1002/acr.24130 32090466

[B23] Skorpen C. G. Hoeltzenbein M. Tincani A. Fischer-Betz R. Elefant E. Chambers C. (2016). The EULAR points to consider for use of antirheumatic drugs before pregnancy, and during pregnancy and lactation. Ann. Rheum. Dis. 75 (5), 795–810. 10.1136/annrheumdis-2015-208840 26888948

[B24] Stergiakouli E. Thapar A. Smith G. D. (2016). Association of acetaminophen use during pregnancy with behavioral problems in childhood: evidence against confounding. JAMA Pediatrics 170, 964–970. 10.1001/jamapediatrics.2016.1775 27533796 PMC5300094

[B25] Thomas R. Sanders S. Doust J. Beller E. Glasziou P. (2015). Prevalence of attention-deficit/hyperactivity disorder: a systematic review and meta-analysis. Pediatrics 135, e994–e1001. 10.1542/peds.2014-3482 25733754

[B26] Urowitz M. Gladman D. MacKinnon A. Ibanez D. Bruto V. Rovet J. (2008). Neurocognitive abnormalities in offspring of mothers with systemic lupus erythematosus. Lupus 17, 555–560. 10.1177/0961203308089326 18539709

[B27] Vinet É. Pineau C. A. Clarke A. E. Scott S. Fombonne É. Joseph L. (2015). Increased risk of autism spectrum disorders in children born to women with systemic lupus erythematosus: results from a large population‐based cohort. Arthritis Rheumatol. 67, 3201–3208. 10.1002/art.39320 26315754

[B28] Yengej F. A. Y. van Royen-Kerkhof A. Derksen R. H. Fritsch-Stork R. D. (2017). The development of offspring from mothers with systemic lupus erythematosus. A systematic review. Autoimmun. Rev. 16, 701–711. 10.1016/j.autrev.2017.05.005 28479488

